# Letter in response to Tarallo *et al* 2022

**DOI:** 10.1308/rcsann.2022.0089

**Published:** 2022-10-20

**Authors:** P Garfjeld Roberts

**Affiliations:** Oxford Orthopaedic Simulation and Education Centre, UK

Dear Professor Rogers

It was heartening to see an evidence-based approach to surgical training in the most recent article by Tarallo *et al* as surgical simulation research matures as a scientific discipline.^1^ However, there are important problems with chosen metrics and how they are analysed. This matters because use of inappropriate statistics to assess performance or competence could have serious consequences if applied to surgery.

First, the CUSUM is an industrial process control method to monitor steady-state performance: the test statistic detects when performance deviates from an ‘in-control’ to ‘out-of-control’ state. CUSUM has been coopted to investigate learning curves,^2^ but because trainees performance must be assumed ‘out-of-control’ to begin with (hence the need for assessment), it violates the assumptions of the test.^3^ Accepting these violations leads to the unusual phenomenon seen in Figure 1 of the paper where the trainees are in-, then out-of-, then in-control again, and the senior surgeon eventually becomes ‘out-of-control’. Fortunately for the patients, they are ‘out-of-control’ in a ‘good way’, passing the lower CUSUM boundary (case 48 in Centre 1 and case 45 in Centre 2). What the authors want is the LC-CUSUM (learning curve CUSUM): a related concept but essentially opposite test where trainees have to demonstrate they are ‘in-control’.^3^

Second, operative time is a process outcome that, in itself, does not represent surgical skill and provides a perverse incentive for trainees.^4,5^ All other aspects being equal (the authors diligently report the important inputs for a successful procedure and complications in their methods section), you could compare two procedures by time taken; it should be the last thing we assess, not the first. All those involved in training should be mindful of Goodhart’s Law (‘When a measure becomes a target, it ceases to be a good measure’), especially if it is analysed incorrectly.
